# Dark-responsive BGH2 and light-responsive BPG4: Taming the GLK1/2 master transcription factors for etioplast and chloroplast homeostasis

**DOI:** 10.1093/plcell/koaf190

**Published:** 2025-08-05

**Authors:** Jiajun Wang

**Affiliations:** Assistant Features Editor, The Plant Cell, American Society of Plant Biologists; School of Life Sciences, Xiamen Key Laboratory of Plant Genetics, Xiamen University, Xiamen 361102, China

Chlorophyll is the central pigment of photosynthesis in plants, capturing light energy that is converted into chemical energy that fuels plant growth and development. In angiosperms, chlorophyll biosynthesis depends on nuclear-encoded genes, collectively referred to as photosynthesis-associated nuclear genes (Wang and Grimm 2015). Chlorophyll biosynthesis is essential for chloroplast development. However, excessive accumulation of the intermediate protochlorophyllide (Pchlide) can lead to photooxidative damage, compromising chloroplast structure and function (Vedalankar and Tripathy 2024). Meanwhile, chlorophyll is incorporated into the photosynthetic complexes, photosystems I and II, where it stabilizes the protein assemblies and promotes proper thylakoid membrane folding, thereby ensuring the integrity of the chloroplast (Wang and Grimm 2015).

*Brz-insensitive-Pale Green 4* (*BPG4*), also known as *Repressor of Photosynthetic Genes 2* (*RPGE2*) and *Pseudo-Etiolation in Light 1* (*PEL1*), represses chlorophyll *a* and *b* biosynthesis and maintains chloroplast homeostasis by interacting with GOLDEN2-LIKE1/2 (GLK1/2) transcription factors. This interaction inhibits the binding of GLK1/2 to the promoters of chlorophyll biosynthesis–related genes, thereby suppressing their transcriptional activation (Tachibana et al. 2024). The BPG4/RPGE/PEL family includes 4 members: BPG4/RPGE2/PEL1, BGH1/RPGE3/PEL3, BGH2/RPGE1/PEL2, and BGH3/RPGE4/PEL4. Although overexpression of *BGH2*, similar to *BPG4*, significantly reduces chlorophyll content (Kim et al. 2016, 2023; Tachibana et al. 2024), *bgh2* mutants show no significant change in chlorophyll content (Kim et al. 2016; Han et al. 2024; Tachibana et al. 2024). Interestingly, unlike *BPG4*, whose transcription is light induced, *BGH2* is mainly expressed in dark-grown seedlings (Kim et al. 2016). Whether *BGH2* regulates the synthesis of chlorophyll precursors in darkness to facilitate the adaptation of etiolated seedlings to light remains to be investigated. In new work, Ryo Tachibana and collaborators (2025) explore the function of *BGH2* in Arabidopsis (*A. thaliana*).

The authors showed that *BGH2* is highly expressed in etiolated seedlings and is rapidly suppressed by light, whereas *BPG4* is barely expressed in etiolated seedlings and is induced by light. To explore the function of *BGH2*, cotyledon greening rates and Pchlide levels were measured in wild type, *bgh2* mutant, and *BGH2*-overexpressing plants during dark-to-light transition. Etiolated *bgh2* mutants (grown in darkness for 6 to 8 d) displayed significantly lower greening rates than the wild type, whereas *bpg4* mutants exhibited no difference. Additionally, *bpg4 bgh2* double mutants did not enhance the low-greening phenotype of bgh2. In dark-grown *bgh2* mutants, transcript levels of key Pchlide biosynthesis genes (*HEMA1*, *GUN4*, *CHLH/GUN5*, and *CHL27*) were significantly upregulated while downregulated in *BGH2*-overexpressing lines. Consistently, total Pchlide contents in etiolated *bgh2* mutants were significantly higher than those in the wild type, with increases in POR-bound photoactive Pchlide (which converts to chlorophyllide upon light exposure without generating harmful singlet oxygen) and nonphotoactive free Pchlide (a photosensitizer that produces singlet oxygen and induces cell death). Notably, the ratio of nonphotoactive to photoactive Pchlide was significantly increased in *bgh2* mutants and decreased in overexpression lines as compared with the wild type. During the dark-to-light transition, nonphotoactive Pchlide levels, singlet oxygen production, and cell death were all increased in *bgh2* mutants. These results suggest that *BGH2* represses excessive Pchlide accumulation in the dark, thereby protecting etiolated seedlings from photooxidative damage during de-etiolation.

To explore the molecular mechanism of BGH2-mediated repression of Pchlide biosynthesis, the authors further analyzed the biochemical and genetic relationships between BGH2 and GLK1/2. Although GLK1 and GLK2 transcripts and protein levels are low in the dark, BGH2 and GLK1 still interact in etiolated seedlings. In contrast to the *bgh2*, dark-grown *glk1 glk2* mutants exhibited significantly lower Pchlide levels and higher greening rates than the wild type. The *bgh2 glk1 glk2* triple mutants largely rescued the *bgh2* phenotypes, including elevated expression of *HEMA1*, *GUN4*, and *CHLH/GUN5*; excessive Pchlide accumulation; and low greening rates. This demonstrates that BGH2 represses GLK1/2 transcriptional activation of key Pchlide biosynthesis genes in the dark and facilitates the adaptation of etiolated seedlings to light.

In darkness, PIF1, PIF3, PIF4, and PIF5 bind to the promoters of *BGH2* and *BPG4*, promoting BGH2 expression in the dark and BPG4 expression under red light (Kim et al. 2016). To dissect the transcriptional regulation responsible for BGH2 and BPG4 expression under dark and light conditions, the authors used electrophoretic mobility shift assays and dual-luciferase assays. The results showed that PIF4 directly binds to 2 G-box elements in the BGH2 and BPG4 promoters and primarily activates their expression via the G-box proximal to the ATG. Importantly, *BGH2* overexpression rescued the low-greening phenotype of *pif1* and *pif3* mutants, genetically placing *BGH2* downstream of *PIFs* in regulating greening. Given that light promotes PIF degradation, light-induced *BPG4* expression depends on additional factors beyond PIFs. The authors found that GLK1/2 directly bind to the *BPG4* promoter to activate its transcription. Since BPG4 interacts with GLK1/2 and suppresses their transcriptional activity, this forms a negative feedback loop that fine-tunes chloroplast homeostasis under light conditions.

In summary, this work, with previous studies, demonstrates that *BGH2* transcription is induced by PIFs in darkness, leading to suppression of GLK1/2 activity, repressing excessive accumulation of Pchlide in etiolated seedings, thereby preventing photobleaching and cell death during the dark-to-light transition. In contrast, *BPG4* expression is light induced by GLK1/2 and PIFs, forming a BPG4-GLK1/2 negative feedback loop that maintains chloroplast homeostasis under light (Fig.).

**Figure.**
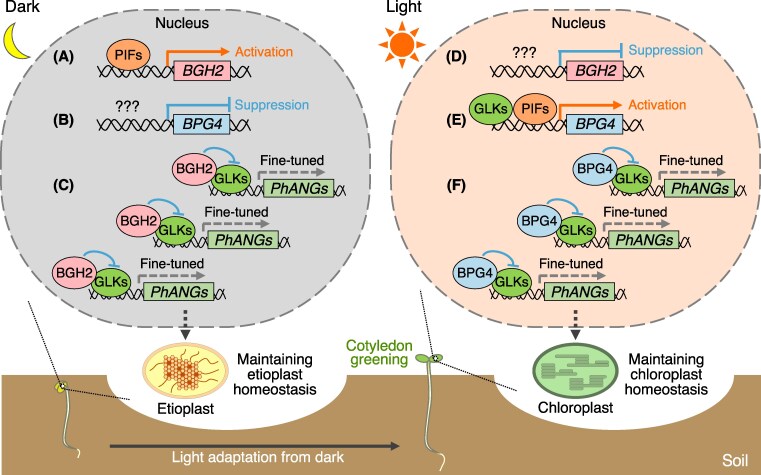
Working model of BGH2- and BPG4-mediated regulation of etioplast and chloroplast homeostasis under dark and light conditions. **A)** In etiolated seedlings grown in the dark, stabilized PIFs activate *BGH2* expression. **C)** BGH2 inhibits GLK activity to fine-tune the expression of photosynthesis-associated nuclear genes (PhANGs), maintaining suitable Pchlide content and etioplast homeostasis in the dark. **E)** In the light, *BPG4* is activated by PIFs. GLKs also **E)** directly enhance *BPG4* expression, **F)** preventing excessive GLK accumulation. **F)** BPG4 contributes to maintaining adequate chlorophyll levels and chloroplast homeostasis in the light. The suppressive mechanisms of **B)**  *BPG4* expression in the dark and **D)**  *BGH2* expression in the light are unclear. Reprinted from Tachibana et al. (2025; Figure 9).

## Recent related articles in *The Plant Cell*

- Wang et al. (2024) illuminated that wild type DcRPGE1 inhibits carotenoid biosynthesis by interfering with DcAPRR2-mediated activation of *DcPSY1/2* and *DcLCYE*, while a mutation in domesticated carrots abolishes this repression, enabling carotenoid accumulation.
- Pei et al. (2025) revealed that the AP2/ERF transcription factor *SlDEAR1*, regulated by light and the upstream factor SlHY5, acts as a bifunctional regulator in tomato fruits by promoting chlorophyll biosynthesis through activation of *SlPOR1* and suppressing degradation via recruitment of the TPL2-HDA1/3 complex to repress *SlSGR1*.
- Rong et al. (2025) demonstrated that LTD synchronizes chlorophyll biosynthesis and LHCP transport in Arabidopsis by interacting with and stabilizing key enzymes (Mg-protoporphyrin methyltransferase and MgPME cyclase) and directly binding MgPME, thereby ensuring proper chlorophyll insertion into LHCPs and preventing phototoxicity.

## Data Availability

No new data were generated or analysed in support of this.

## Dive Curated Terms

The following phenotypic, genotypic, and functional terms are of significance to the work described in this paper:

GLK2 Gramene: AT5G44190
GLK2 Araport: AT5G44190

GUN4 Gramene: AT3G59440
GUN4 Araport: AT3G59440

HEMA1 Gramene: AT1G58290
HEMA1 Araport: AT1G58290

GLK1 Gramene: AT2G20570
GLK1 Araport: AT2G20570

chlorophyll CHEBI: CHEBI:28966

BGH2 Gramene: AT5G02580
BGH2 Araport: AT5G02580

BPG4 Gramene: AT3G55240
BPG4 Araport: AT3G55240

BGH1 Gramene: AT3G28990
BGH1 Araport: AT3G28990

BGH3 Gramene: AT1G10657
BGH3 Araport: AT1G10657

## References

[koaf190-B1] Han Y, Li F, Wu Y, Wang D, Luo G, Wang X, Wang X, Kuang H, Larkin RM. PSEUDO-ETIOLATION IN LIGHT proteins reduce greening by binding GLK transcription factors. Plant Physiol. 2024:194(3):1722–1744. 10.1093/plphys/kiad641 38051979

[koaf190-B2] Kim K, Jeong J, Kim J, Lee N, Kim ME, Lee S, Chang Kim S, Choi G. PIF1 regulates plastid development by repressing photosynthetic genes in the endodermis. Mol Plant. 2016:9(10):1415–1427. 10.1016/j.molp.2016.08.007 27591813

[koaf190-B3] Kim N, Jeong J, Kim J, Oh J, Choi G. Shade represses photosynthetic genes by disrupting the DNA binding of GOLDEN2-LIKE1. Plant Physiol. 2023:191(4):2334–2352. 10.1093/plphys/kiad029 36702576 PMC10069884

[koaf190-B4] Pei Y, He X, Xue Q, Deng H, Xu W, Yang C, Wu M, Wang W, Tang W, Niu W, et al The bifunctional transcription factor DEAR1 oppositely regulates chlorophyll biosynthesis and degradation in tomato fruits. Plant Cell. 2025:37(7):koaf167. 10.1093/plcell/koaf167 40577586

[koaf190-B5] Rong L, An J, Chen X, Wang C, Wu J, Wang P, Zheng Y, Wang X, Chai X, Li W, et al LTD coordinates chlorophyll biosynthesis and LIGHT-HARVESTING CHLOROPHYLL A/B-BINDING PROTEIN transport. Plant Cell. 2025:37(4):koaf068. 10.1093/plcell/koaf068 40138376 PMC11979457

[koaf190-B6] Tachibana R, Abe S, Marugami M, Yamagami A, Akema R, Ohashi T, Nishida K, Nosaki S, Miyakawa T, Tanokura M, et al BPG4 regulates chloroplast development and homeostasis by suppressing GLK transcription factors and involving light and brassinosteroid signaling. Nat Commun. 2024:15(1):370. 10.1038/s41467-023-44492-5 38191552 PMC10774444

[koaf190-B7] Tachibana R, Akema R, Yoshihara A, Ujihara C, Nishida K, Ri S, Yamagami A, Miyakawa T, Kobayashi K, Tanaka R, et al Dark-inducible BGH2 suppresses GLK transcription factors and maintains plastid homeostasis to promote light adaptation. Plant Cell. 2025:37(8):koaf180. 10.1093/plcell/koaf180 40694618

[koaf190-B8] Vedalankar P, Tripathy BC. Light dependent protochlorophyllide oxidoreductase: a succinct look. Physiol Mol Biol Plants. 2024:30(5):719–731. 10.1007/s12298-024-01454-5 38846463 PMC11150229

[koaf190-B9] Wang P, Grimm B. Organization of chlorophyll biosynthesis and insertion of chlorophyll into the chlorophyll-binding proteins in chloroplasts. Photosynth Res. 2015:126(2–3):189–202. 10.1007/s11120-015-0154-5 25957270

[koaf190-B10] Wang YG, Zhang YM, Wang YH, Zhang K, Ma J, Hang JX, Su YT, Tan SS, Liu H, Xiong AS, et al The Y locus encodes a REPRESSOR OF PHOTOSYNTHETIC GENES protein that represses carotenoid biosynthesis via interaction with APRR2 in carrot. Plant Cell. 2024:36(8):2798–2817. 10.1093/plcell/koae111 38593056 PMC11289637

